# Acceptance of Organs from Deceased Donors With Resolved or Active SARS-CoV-2 Infection: A Survey From the Council of Europe

**DOI:** 10.3389/ti.2024.13705

**Published:** 2024-11-21

**Authors:** Maddalena Peghin, Elena Graziano, Maria De Martino, Maria Luisa Balsamo, Miriam Isola, Marta López-Fraga, Massimo Cardillo, Giuseppe Feltrin, Beatriz Domínguez-Gil González, Paolo Antonio Grossi, The COVIDonors COE Study Group

**Affiliations:** ^1^ Infectious and Tropical Diseases Unit, Department of Medicine and Surgery, University of Insubria-Azienda Socio Sanitaria Territoriale (ASST)-Sette Laghi, Varese, Italy; ^2^ Division of Medical Statistic, Department of Medicine, University of Udine, Udine, Italy; ^3^ European Directorate for the Quality of Medicines and HealthCare (EDQM), Strasbourg, France; ^4^ Italian National Center for Transplantation (CNT), Rome, Italy; ^5^ Organización Nacional de Trasplantes, Ministerio de Sanidad, Madrid, Spain

**Keywords:** DDI, donor, recipient, Sars-CoV-2, COVID-19, donor derived infections

## Abstract

SARS-CoV-2 infection represents a new challenge for solid organ transplantation (SOT) with evolving recommendations. A cross-sectional survey was performed (February–June 2024) to describe practices among Member States of the Council of Europe (COE) on the use of organs from deceased donors with resolved or active SARS-CoV-2 infection. Overall, 32 out of 47 Member States with a transplant program participated in the study. Four (12.5%) countries did not use organs from deceased donors either with resolved or with active SARS-CoV-2 infection and 8 (25%) countries accepted organs only from deceased donors with resolved SARS-CoV-2 infection. Donor evaluation for SARS-CoV-2 included universal screening with standard PCR testing on respiratory specimens generally (61.4%) performed within 24 h prior to organ recovery. Further microbiological, immunological and radiological investigations varied. Most waitlisted patients receiving organs from a deceased donor with active (94.5%) or resolved (61.5%) SARS-CoV-2 infection were preferred to have natural, vaccine-induced or hybrid SARS-CoV-2 immunity. Most countries did not require recipients to undergo specific anti-SARS-CoV-2 treatment as pre-exposure (0%), post-exposure prophylaxis (15.4%) or modification of immunosuppression regimen (24%). This study highlights similarities and heterogeneities in the management of SARS-CoV-2 positive donors between COE countries, and a potential to safely expand donors’ pool.

## Graphical Abstract

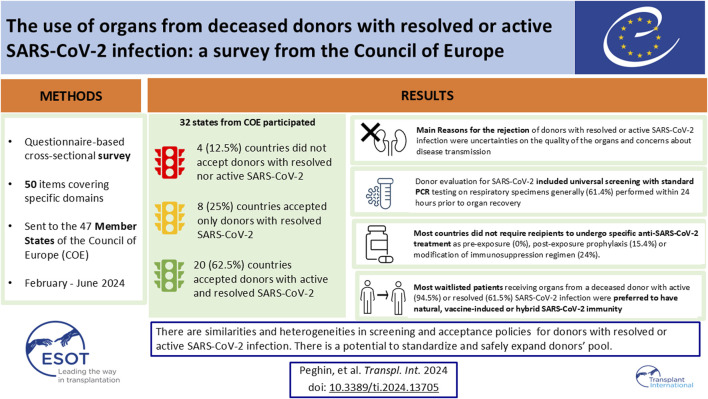

## Introduction

The increasing gap between patients on the waiting list and organ availability has led to the use of organs from donors with well-known and emerging infections, supported by the improvement of risk mitigation strategies to avoid donor derived infections [1].

At the beginning of the severe acute respiratory syndrome coronavirus 2 (SARS-CoV-2) pandemic, uncertainties existed regarding the route of transmission of SARS-CoV-2; this created pressure on transplant systems to recommend universal donor screening and advise against solid organ transplantation (SOT) from donors testing positive for SARS-CoV-2 [2]. Such restrictive policies resulted in the loss of a significant number of lifesaving and life-enhancing organs [3]. Based on growing evidence of the biology of SARS-CoV-2, along with the availability of effective vaccines and new treatment options, these recommendations have been challenged and transplant systems worldwide have adopted various policies and regularly updated guidance for organ acceptance and recipient management [2, 4, 5].

This manuscript describes current transplantation practices in Member States of the Council of Europe (COE) with regards to the use of organs from deceased donors with resolved or active SARS-CoV-2 infection.

## Materials and Methods

A questionnaire-based cross-sectional survey was developed based on a scoping review of the literature and was independently reviewed by four study investigators (PAG, MP, EG, MLB) who were part of an expert panel. A definitive questionnaire with 50 items covering specific domains was based on the full consensus of the investigators. The survey was sent by the European Directorate for the Quality of Medicines and HealthCare (EDQM) of the Council of Europe (COE) to Member States of the COE. The survey was hosted on a cloud-based software (SurveyMonkey^®^, San Mateo, CA, United States) between February and June 2024. Member States who were willing to participate were included.

A deceased donor with resolved SARS-CoV-2 infection was defined as a donor who died after the resolution of symptoms and had viral clearance documented by a negative SARS-CoV-2 RT-PCR or antigenic test in respiratory samples. A deceased donor with active SARS-CoV-2 infection was defined as a donor who died and had a positive SARS-CoV-2 RT-PCR or antigenic test in respiratory samples.

The reference European committee on organ transplantation of the COE (CD-P-TO) approved the study and all procedures were in accordance with established ethical standards (TO129).

### Statistical Analysis

A descriptive analysis was performed. Continuous variables are expressed as the median and range. All proportions were calculated as percentages of patients with available data. Data analyses were performed with Stata 17 software.

## Results

### General COE Practices

**FIGURE 1:**
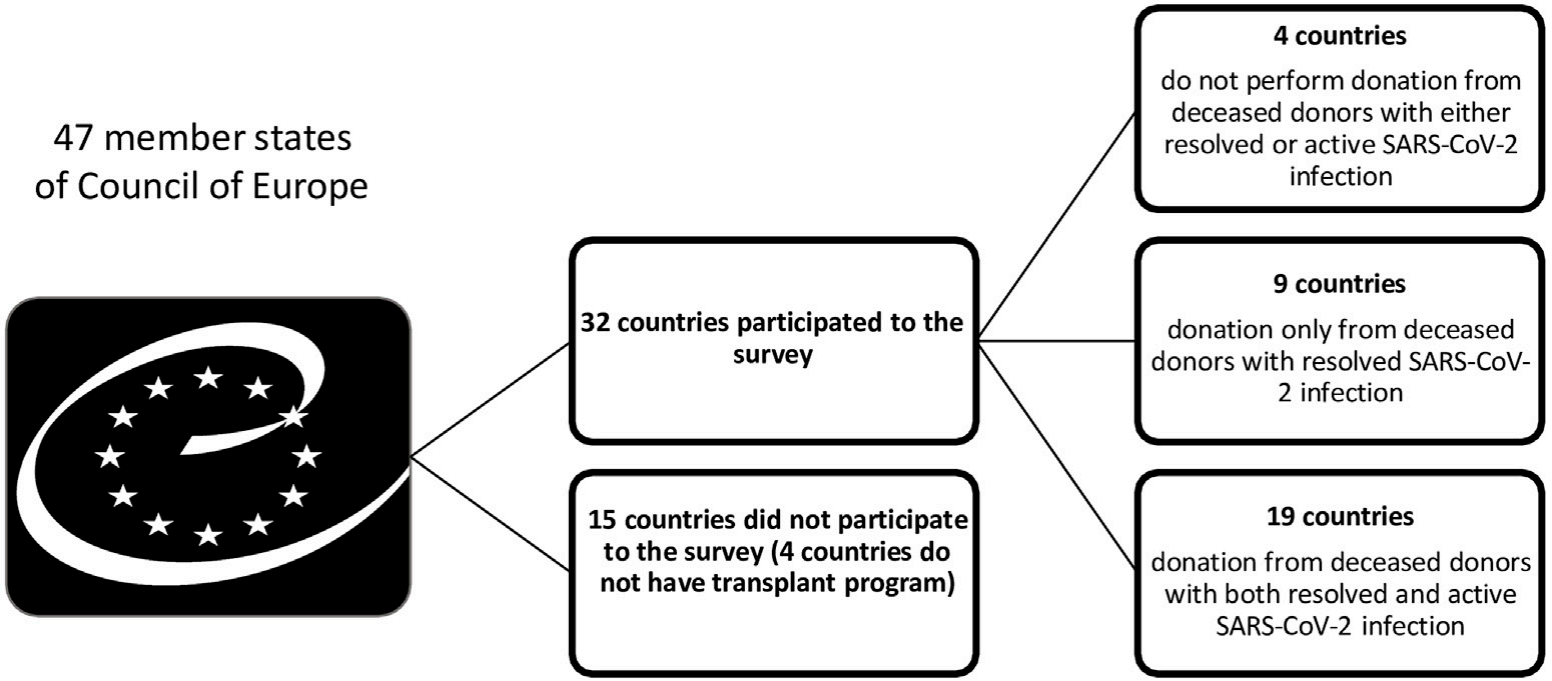
Use organs from deceased donors with resolved or active SARS-CoV-2 infection across Council of Europe Member States.

**FIGURE 2:**
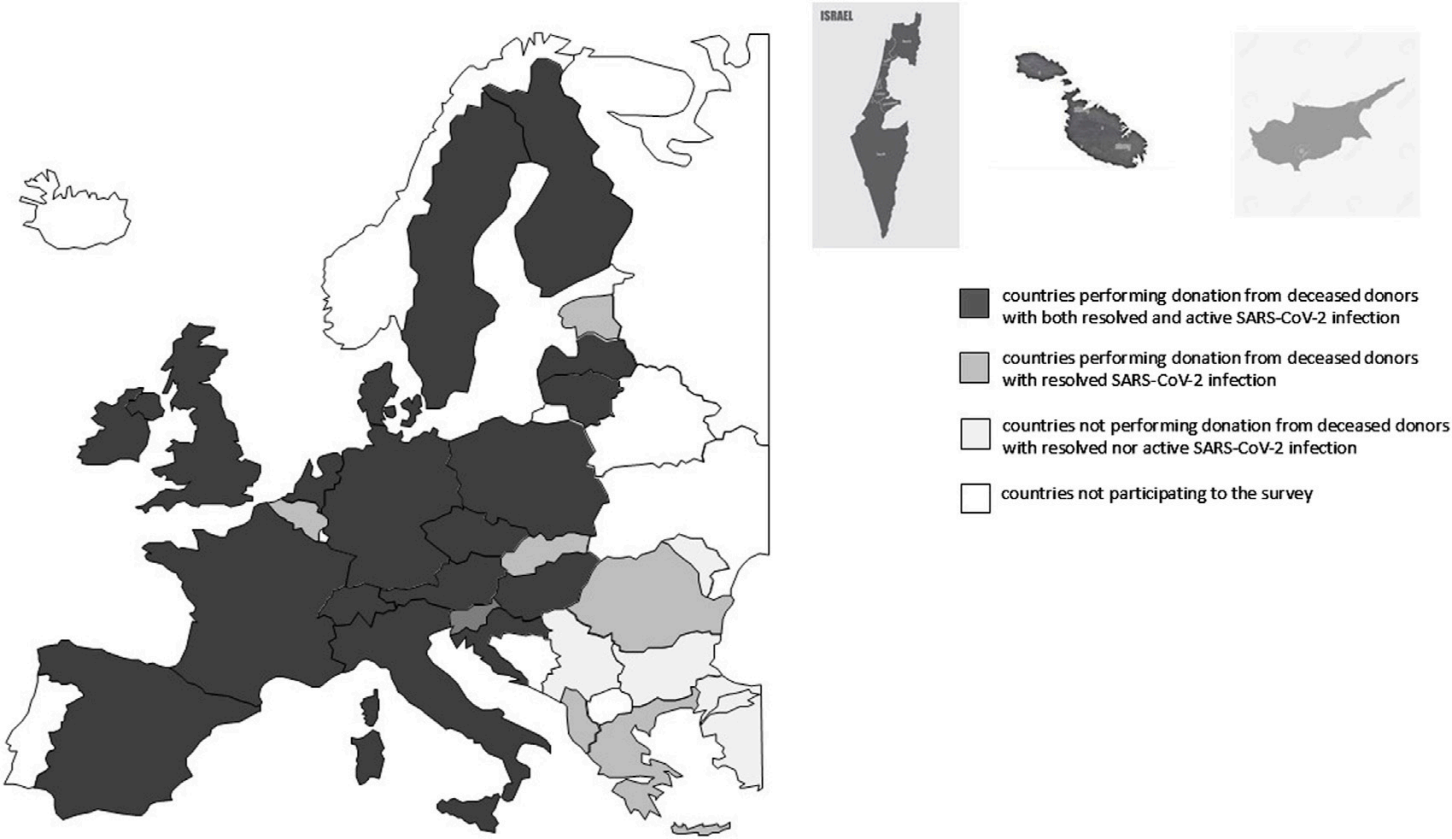
European map of countries participating to the study.

Thirty-two out of 47 Member States of the COE participated in the study. Four countries do not have an active transplant program. Around 4 (12.5%) countries did not use organs from deceased donors with either resolved or active SARS-CoV-2 infection and 8 (25%) countries did not accept organs from deceased donors with active SARS-CoV-2 infection because of uncertainties regarding the risk of SARS-CoV-2 transmissibility (Figures 1, 2).

### Use of Organs From Deceased Donors With Resolved SARS-CoV-2 Infection

Overall, 28 (87.5%) countries accepted organs from deceased donors with resolved SARS-CoV-2 infection. This transplantation practice was active for a median of 26.2 months (range 17.3–48.8 months), from June 2020 to June 2024. Organs accepted for transplantation from donors with resolved SARS-CoV-2 infection included kidney (28/28, 100%), liver (27/28; 96.4%), heart (25/28; 89.3%), pancreas (21/28; 75%), lung (18/28; 64.3%) and bowel (17/28; 60.7%).

Organ procurement was allowed from deceased donors who died at least an average of 11.4 days (range 0–30 days) after resolution of symptoms for non-lung transplant and an average of 14.5 days (range 10–21 days) for lung transplant. All countries required the donor to be asymptomatic at the time of procurement and to have viral clearance documented by a negative SARS-CoV-2 PCR in nasopharyngeal swab (NPS) and/or lower respiratory tract (LRT) sample. The main reasons for refusal of organs from donors with resolved SARS-CoV-2 infection are listed in Table 1.

**TABLE 1:** Reasons for the rejection of donors with resolved or active SARS-CoV-2 infection.

	Donors with active COVID-19	Donors with resolved COVID-19
	n/N, % (n = 18)	n/N, % (n = 21)
Uncertainties on the quality of the organs	9 (50)	14 (66.7)
Concerns about disease transmission	11 (61.1)	5 (23.8)
Severity of COVID-19	8 (44.4)	1 (4.8)
Infectious diseases specialist’s decision	5 (27.8)	3 (14.3)
Thrombotic complications	3 (16.7)	2 (9.5)
Inflammatory activity	1 (5.5)	2 (9.5)
Organ damage	2 (11.1)	8 (38.1)

### Use of Organs From Deceased Donors With Active SARS-CoV-2 Infection

Overall, 20 (62.5%) countries accepted organs from deceased donors with active SARS-CoV-2 infection. This policy was active on average for 24.1 months (range 17.1–48.1 months), between November 2020 and June 2024. Organs considered for donation from patients with active SARS-CoV-2 infection included kidney (19/20; 95%), heart (18/20; 90%), liver (19/20; 95%), pancreas (15/20; 75%), lung (2/20; 10%) and bowel (6/20; 30%). All countries required the donor to be asymptomatic or mildly symptomatic for SARS-CoV-2 infection to allow donation. The main reasons for refusal of organs from donors with active SARS-CoV-2 infection are listed in Table 1.

### Recipients With Active or Resolved SARS-CoV-2 Infection

In general, recipients with active SARS-CoV-2 infection were not allowed to receive organs from donors with active SARS-CoV-2 infection (11/21; 52.4%), except by case-by-case infectious disease evaluation (8/21; 38.1%). Only one country allowed transplantation of individuals with active infection routinely.

Patients with recently resolved SARS-CoV-2 positivity could be re-entered on the transplant waiting lists in most cases after resolution of symptoms and documented virological cure with negative SARS-CoV-2 PCR (19/24; 79.2%). The minimum duration of recipient symptoms from SARS-CoV-2 onset to allow transplantation averaged 19 days (range 0–90).

### Screening and Eligibility of Waitlisted Patients

Overall, most national protocols (27/32; 84.4%) recommend waitlisted patients to be vaccinated with a median of 3 doses (range 2–5) of SARS-CoV-2 vaccine. SARS-CoV-2 vaccination was mandatory for waitlisted patients in 5 out of 32 (15.6%) countries. Based on current protocols, SARS-CoV-2 IgG measurement was not routinely performed for most waitlisted patients before transplantation (21/32; 62.6%). SARS-CoV-2 virus-specific cell-mediated immunity was regularly determined before transplantation only in three countries.

Most waitlisted patients were allowed to receive organs from a deceased donor with active (94.5%) or resolved (61.5%) SARS-CoV-2 infection, only if they met specific conditions. Mandatory criteria are listed in Table 2.

**TABLE 2:** Conditions required by potential recipients to receive organs from donors with resolved or active SARS-CoV-2 infection.

	Donors with active COVID-19	Donors with resolved COVID-19
	n/N, % (n = 18)	n/N, % (n = 26)
History of resolved COVID-19	7 (38.9)	5 (19.2)
Full vaccination (at least 3 doses)	10 (55.5)	10 (38.5)
Documented immunological response (seroconversion and/or virus-specific cell-mediated immunity)	3 (16.7%)	2 (7.7)
Life-threatening organ dysfunction and low probability of a suitable and timely non-infected donor	4 (22.2)	2 (7.7)
None	1 (5.5%)	10 (38.5)
Case-by-case evaluation	5 (27.8%)	3 (11.5)

The majority (17/25; 68%) of national protocols had a specific recipient informed consent required for patients receiving organs from a deceased donor with resolved or active SARS-CoV-2 infection.

### Standard Donor SARS-CoV-2 Screening

Current strategies for donor screening for SARS-CoV-2 included evaluation by SARS-CoV-2 PCR. Testing recommendations for non- lung, non-bowel donation included collection of samples from the LRT and NPS (10/29; 34.5%) and from the LRT or NPS (13/29; 44.8%). Most countries performed the assay (18/29; 62.1%) within 1 day before organ recovery.

Regarding lung and bowel donation, routine recommendations were to collect samples from the LRT and NPS (18/26; 69.2%) or only from the LRT (4/26; 15.4%). Most countries performed the assay (17/28; 60.7%) within 1 day before to organ recovery.

### Specific Donor SARS-CoV-2 Screening for Deceased Donors With Active or Resolved SARS-CoV-2 Infection

SARS-CoV-2 cycle threshold (Ct) values were required and considered in decision-making processes in 9 countries (11/28; 39.3%) and on a case-by-case basis in 3 countries (3/28; 10.7%).

SARS-CoV-2 IgG measurement and/or SARS-CoV-2 virus-specific cell-mediated immunity was not routinely performed in most donors (27/30; 90%).

SARS-CoV-2 PCR was performed on samples of donor organ biopsy in two countries (2/26; 7.7%) only for deceased donors with active SARS-CoV-2 infection and in one country (1/26; 3.8%) both for active and resolved SARS-CoV-2 donors. SARS-CoV-2 PCR on preservation fluid and analysis of donor organ quality with biopsy was recommended at time of donation only in one country mainly for research purpose.

Chest imaging with computed tomography (CT) scan was routinely performed both for donors with resolved or active SARS-CoV-2 infection (16/28; 57.2%) and only for active (2/28; 7.1%). No routine CT imaging was usually required in 10 out of 28 (35.7%) countries, except in specific settings.

### Hospital Setting Preventive Measures for Transplantation

During organ procurement and transplant, infection control measures included the use of filtering facemasks (N95, FFP2 and FFP3) (23/28; 82.1%), eye protection (19/28; 67.6%), dedicated operating theatres (7/28; 25%) and standard gloves and gowns (28/28; 100%).

Recipients of organs from donors with resolved SARS-CoV-2 infection were not placed in isolation and were managed per routine in most countries (20/26; 76.9%). Other countries placed recipients in isolation in an individual room in a non-SARS-CoV-2 area (5/26; 19.2%) or considered hospital-specific procedures (1/26; 3.9%).

Recipients of organs from donors with active SARS-CoV-2 were isolated based on local centre protocols (10/17; 58.8%), isolated in an individual room in a general ward (5/17; 29.6%) or in a SARS-CoV-2 area (1/17; 5.9%). Only one country managed the recipients without specific isolation procedures.

Among countries performing lung transplantation from donors with resolved SARS-CoV-2 infection, 77% (14/18) manage recipients after transplantation routinely, while 11% (2/18) place recipients in isolation in an individual room in a non-COVID-19 area but with isolation procedures. Among the two countries performing lung transplantation from active COVID-19 donors, one manages recipients as routinely and the other one did not specify the isolation procedure.

Vaccination was mandatory for healthcare workers in 37.5% (12/32) countries and was required for family members visiting hospitals in one country.

SARS-COV-2 infections related to the organ procurement or transplantation among healthcare workers were not observed in most countries for which data were available (21/21; 100%).

### Treatment Strategies After Transplant

None of the countries recommended routine pre-exposure prophylaxis before transplant for recipients of organs obtained from donors with resolved or active SARS-CoV-2 infection. Post-exposure prophylaxis was not suggested after transplant for recipients of organs with resolved and/or active SARS-CoV-2 infection in most COE countries (22/26; 84.6%). With regards to lung transplantation, most countries performing lung transplantation from donors with resolved SARS-CoV-2 infection (16/18; 88%) do not perform any post-exposure prophylaxis on recipients, as well as one out of the two countries performing lung transplantation from active COVID-19.

Immunosuppression regimens were not routinely changed after transplantation in most countries (19/25; 76%). Within the 10 countries performing transplants for recipients with active SARS-CoV-2 infection, 1 (10%) recommended specific SARS-CoV-2 treatment after transplant and 2 (20%) suggested immunosuppression regimen modification.

### Follow-Up After Transplant and Recipient Outcome

After transplantation, all recipients were routinely monitored clinically, about half virologically with periodic SARS-COV-2 PCR in respiratory samples (46.3%, 13/28) and only two immunologically (7.1%, 2/28) with SARS-COV-2 serological testing. No donor derived SARS-CoV-2 infection was described in any country.

Survey results are summarized in Supplementary Tables S1–S18.

## Discussion

This survey provides an overview of the policies and real-life use of SARS-CoV-2 positive donors for SOT in 32 countries across Europe. To our knowledge, this is the first international assessment using a standardized questionnaire and providing detailed information about the management practice of organs obtained from deceased donors with resolved or active SARS-CoV-2 infection.

Since the beginning of the pandemic, SARS-CoV-2 infection caused discarding many organs while efforts to maintain SOT activities were being made worldwide [2, 3]^.^ Through our survey, we found increasing support to the acceptance of grafts from deceased donors SARS-CoV-2 positive in Member States of the COE. However, we also identified four countries where organs from deceased donors, either with resolved or active SARS-CoV-2 infection, are not used, as well as eight countries where organs from deceased donors with active SARS-CoV-2 infection are not accepted for SOT.

On the basis of this survey and recent worldwide experience, transplantation of non-lung and non-bowel organs from donors with active SARS-CoV-2 infection is considered safe, without evidence of SARS-CoV-2 transmission. In addition, good short-term outcomes, in terms of graft loss and mortality, have been observed and confirmed in our survey [6–10]. This should prompt more countries to reconsider their policies with regards to the use of organs from SARS-CoV-2 positive donors. However, SARS-CoV-2 infection could potentially lead to adverse outcomes in the long-term, likely due to subclinical endothelial dysfunction, hypercoagulability and organ injury in potential donors; pertinent data on this point are limited from both this COE survey and other available studies [11]. Similar patient and graft survival has been reported among kidney and liver transplant recipients over 1 year after transplantation, regardless of donor SARS-CoV-2 infection status [8, 12–14]. Nonetheless, an Italian study with 1 year follow-up found significantly higher rates of hepatic artery thrombosis among recipients of liver grafts from SARS-CoV-2 positive compared with SARS-CoV-2 negative donors [11, 15]. Moreover, an increase in 6- and 12-month mortality has been observed among recipients of hearts obtained from donors with active SARS-CoV-2 infection compared with recipients of hearts from donors with no SARS-CoV-2 infection or with a history of resolved SARS-CoV-2 infection [14]. Further research is needed to evaluate the long-term evolution of recipients from SARS-CoV-2-positive donors, particularly for vascular complications, and to define a more tailored approach to the donor pool.

The use of lung from COVID-19 positive donors is being explored in two COE countries. Recent series have confirmed that lungs from donors with a positive SARS-CoV-2 PCR might be successfully used with cautious donor selection with comparable early post-transplant outcomes to lung allografts from COVID-19-negative donors [10, 14, 16–18]. Lung donor selection include asymptomatic status, high Ct levels (>30–35) and symptom onset or SARS-CoV-2 test positivity older than 20 days [10, 14, 16–18]. Of note that high Ct levels tend to correlate with culture negativity, but Ct values are not available on many platforms and, when obtained, such values may not be comparable between different platforms and laboratories [2]. Further analysis with longer follow-ups is warranted to determine the safety of utilization of COVID-positive donor lungs.

Current strategies for donor evaluation for SARS-CoV-2 infection include universal microbiologic screening with standard SARS-CoV-2 PCR testing, mostly performed within 24 h prior to organ recovery. In keeping with international recommendations, all but one country considered LRT specimen mandatory for lung donation, on the basis of previous unexpected SARS-CoV-2 donor-derived infection in lung recipients, despite negativity of NPS in the donor [6, 10, 19]. Some experts consider that, due to the impact of SARS-CoV-2 positive testing on organ discard, resource utilization and on the basis of data supporting safety of transplanting SARS-CoV-2 non-lung organs, universal testing of non-lung deceased asymptomatic donors should be reconsidered [20].

SARS CoV-2 PCR testing on grafts was performed in three countries; tissue positivity was recently found to be associated with vascular complications after liver transplantation [15]. Further radiological investigations of donors were performed in ∼65% of countries either per protocol or on an individual basis to assess organ damage. Of note, the interpretation of CT scans is challenging, as abnormal CT images are common in SARS-CoV-2 infected patients, even when asymptomatic [21].

In most COE countries, waitlisted patients receiving organs from a deceased donor with active or resolved SARS-CoV-2 infection were preferred to have natural, vaccine-induced or hybrid SARS-CoV-2 associated immunity; documented immunological response was rarely required, so the impact of vaccination is uncertain. Pre-transplant SARS-CoV-2 vaccination should be strongly favored given the expected improvement of immune responses before transplant, the possible decreased risk of complications when infection occurs and the advancement in the use of organs from SARS-CoV-2-positive donors [22]. The exclusion of patients from the transplant waiting list for declining SARS-CoV-2 vaccination was performed in about 15% of COE countries and represents a controversial ethical topic [22].

Wide variability exists in transplant practices for SARS-CoV-2 positive candidates for transplantation. The excellent graft and patient post-transplant outcomes presented in recent series favor an individualized approach [23].

Overall, clinical monitoring of recipients of organs from SARS-CoV-2 positive donors was routine across COE countries. Anti-SARS-CoV-2 pre-exposure or post -exposure prophylaxis was not required, and standard immunosuppression was generally recommended.

Healthcare workers were identified early in the pandemic to be at higher risk of contracting SARS-CoV-2 infection [24]. Optimal infection control measures were recommended in COE countries to perform SOT from SARS-CoV-2 positive donors, with no reported infections related to the organ procurement or transplantation among healthcare professionals.

Our study has several limitations. Firstly, it is a survey, which does not allow to establish any causative link or consensus statement, but may serve as a basis for further studies and protocols. Secondly, almost one-third of the COE countries did not join the survey and some of them did not fulfill all the questions, introducing a potential selection bias and providing an unbalanced representation of COE activity. Thirdly, data on infections among healthcare workers were frequently not available. Finally, the SARS-CoV-2 pandemic situation is fluid and requires regular updating of practices and policies. Therefore, the description made in this paper is likely to change in the near future.

In conclusion, our survey provides the first international assessment on the use of organs from SARS-CoV-2 positive donors in Europe. Growing evidence on the absence of transmission and good short-term outcomes with organs from deceased donors with resolved or active SARS-CoV-2 infection has led to increasing support for the acceptance of such grafts in Member States of the COE. Similarities and differences in management across countries are significant. Additional standardized protocols and prospective studies are needed to assess the best management and long-term outcomes of recipients of organs from SARS-CoV-2 infected donors and to define a more nuanced approach towards safely maintain the donor pool.

## Data Availability

The raw data supporting the conclusions of this article will be made available by the authors, without undue reservation.

## Group Member of COVIDonors COE Study Group

Helena Almen (Sweden), Corinne Antoine (France), Martina AnušićJuričić (Croatia), Danica Avsec (Slovenia), Omer Bal (Turkey), Franziska Beyeler (Switzerland), Jurijs Bormotovs (Latvia), Martina Brix-Zuleger (Austria), Paul Calleja (Malta), Martin Chrastina (Slovakia), Jonathan Cohen (Israel), Luc Colenbie (Belgium), Jarosław Czerwiński (Poland), Kristina Grigaite (Lithuania), Alma Idrizi (Albania), Nichon Jansen (Netherlands), Kaj A. Jørgensen (Denmark), Janet Latzel (COE), Marko Lempinen (Finland), Sándor Mihály (Hungary), Antonino Montemurro (Italy), Patricia Montiel (Spain), Petra Novotná (Czech Republic), Jean O’Reilly (Ireland), Virge Pall (Estonia), Alicia Pérez Blanco (Spain), Georgios Papatheodoridis (Greece), Axel Rahmel (Germany), Vladimir Resanovic (Serbia), Andreas Soloukides (Cyprus), Tatiana Timbalari (Moldova), Evelina Tsvetkova (Bulgaria), Ines Ushiro-Lumb (United Kingdom), Guenady Vatachki (Romania), Ilse D. Weinreich (Denmark).

## Abbreviations

COVID-19, coronavirus disease 2019; COE, Council of Europe; Ct, cycle threshold; CT, computed tomography; LRT, lower respiratory tract; NPS, nasopharyngeal swab; PCR, polymerase chain reaction; SARS-CoV-2, Severe Acute Respiratory Syndrome Coronavirus 2; SOT, solid organ transplant.

## References

[1] Peghin M Grossi PA. Donor-Derived Infections in Solid Organ Transplant Recipients. Curr Opin Organ Transpl (2023) 28:384–90. 10.1097/MOT.0000000000001094 PMC10597443 37555801

[2] Peghin M Grossi PA. COVID-19 Positive Donor for Solid Organ Transplantation. J Hepatol (2022) 77(4):1198–204. 10.1016/j.jhep.2022.06.021 35798131 PMC9251900

[3] Aubert O Yoo D Zielinski D Cozzi E Cardillo M Durr M COVID-19 Pandemic and Worldwide Organ Transplantation: A Population-Based Study. Lancet Public Health (2021) 6(10):e709–e719. 10.1016/S2468-2667(21)00200-0 34474014 PMC8460176

[4] Kute VB Fleetwood VA Meshram HS Guenette A Lentine KL. Use of Organs From SARS-CoV-2 Infected Donors: Is It Safe? A Contemporary Review. Curr Transpl Rep (2021) 8(4):281–92. 10.1007/s40472-021-00343-0 34722116 PMC8546195

[5] Romagnoli R Gruttadauria S Tisone G Maria Ettorre G De Carlis L Martini S Liver Transplantation From Active COVID-19 Donors: A Lifesaving Opportunity Worth Grasping? Am J Transpl (2021) 21(12):3919–25. 10.1111/ajt.16823 PMC8653300 34467627

[6] Kaul DR Valesano AL Petrie JG Sagana R Lyu D Lin J Donor to Recipient Transmission of SARS-CoV-2 by Lung Transplantation Despite Negative Donor Upper Respiratory Tract Testing. Am J Transpl (2021) 21(8):2885–9. 10.1111/ajt.16532 PMC8014875 33565705

[7] Dhand A Okumura K Nabors C Nishida S. Solid Organ Transplantation From COVID Positive Donors in the United States: Analysis of United Network for Organ Sharing Database. Transpl Infect Dis (2023) 25(1):e13925. 10.1111/tid.13925 35942924 PMC9538265

[8] Dhand A Gass A John D Kai M Wolf D Bodin R Long-Term and Short-Term Outcomes of Solid Organ Transplantation From Donors With a Positive SARS-CoV-2 Test. Transplantation (2022) 106(8):e384–e385. 10.1097/TP.0000000000004196 35575769 PMC9311281

[9] Goldman JD Pouch SM Woolley AE Booker SE Jett CT Fox C Transplant of Organs From Donors With Positive SARS-CoV-2 Nucleic Acid Testing: A Report From the Organ Procurement and Transplantation Network *ad hoc* Disease Transmission Advisory Committee. Transpl Infect Dis (2023) 25(1):e14013. 10.1111/tid.14013 36694448

[10] Asija R Singh R Paneitz DC Wolfe SB Chukwudi C Michel E Is Transplantation With Coronavirus Disease 2019-Positive Donor Lungs Safe? A US Nationwide Analysis. Ann Thorac Surg (2023) 116:1046–54. 10.1016/j.athoracsur.2023.05.048 37506993

[11] Montiel Villalonga P Martinez-Alpuente I Fernandez-Ruiz M Len O Bodro M Los-Arcos I Transplantation of Organs From SARS-CoV-2-Positive Donors: Preliminary Experience From Spain. Transpl Infect Dis (2023) 25(1):e14008. 10.1111/tid.14008 36659870

[12] Ji M Vinson AJ Chang SH Merzkani M Lentine KL Caliskan Y Patterns in Use and Transplant Outcomes Among Adult Recipients of Kidneys From Deceased Donors With COVID-19. JAMA Netw Open (2023) 6(5):e2315908. 10.1001/jamanetworkopen.2023.15908 37252739 PMC10230314

[13] Wang RX Abu-Gazala S Mahmud N. Posttransplant Outcomes and Trends in Use of COVID-19-Positive Deceased Donor Liver Transplantation. Liver Transpl (2023) 29:1129–33. 10.1097/LVT.0000000000000175 37162163 PMC12036732

[14] Madan S Chan MAG Saeed O Hemmige V Sims DB Forest SJ Early Outcomes of Adult Heart Transplantation From COVID-19 Infected Donors. J Am Coll Cardiol (2023) 81(24):2344–57. 10.1016/j.jacc.2023.04.022 37204379 PMC10191151

[15] Martini S Saracco M Cocchis D Pittaluga F Lavezzo B Barisone F Favorable Experience of Transplant Strategy Including Liver Grafts From COVID-19 Donors: One-Year Follow-Up Results. Transpl Infect Dis (2023) 25:e14126. 10.1111/tid.14126 37585372

[16] Jeon JH Harano T Rodman JCS Sheth M Wightman SC Atay SM Early Outcomes of Lung Transplantation With Lung Allografts From Coronavirus Disease 2019 (COVID-19)-Positive Donors. J Thorac Cardiovasc Surg (2024) 167(6):1955–64.e3. 10.1016/j.jtcvs.2023.08.031 37625616

[17] Organ Procurement and Transplantation Network. Summary of Current Evidence and Information– Donor SARS-CoV-2 Testing and Organ Recovery From Donors With a History of COVID-19 (2024). Available from: https://optn.transplant.hrsa.gov/media/kkhnlwah/sars-cov-2-summary-of-evidence.pdf (Accessed June 15, 2024).

[18] Eichenberger EM Coniglio AC Milano C Schroder J Bryner BS Spencer PJ Transplanting Thoracic COVID-19 Positive Donors: An Institutional Protocol and Report of the First 14 Cases. J Heart Lung Transpl (2022) 41(10):1376–81. 10.1016/j.healun.2022.06.018 PMC9245398 35871114

[19] Saharia KK Ramelli SC Stein SR Roder AE Kreitman A Banakis S Successful Lung Transplantation Using an Allograft From a COVID-19-Recovered Donor: A Potential Role for Subgenomic RNA to Guide Organ Utilization. Am J Transpl (2023) 23(1):101–7. 10.1016/j.ajt.2022.09.001 PMC9833374 36695611

[20] Natori Y Anjan S Hand J. Is It Time to Reconsider Universal Severe Acute Respiratory Syndrome Coronavirus 2 Polymerase Chain Reaction Screening for Asymptomatic Potential Nonlung Solid Organ Transplant Donors? Am J Transpl (2024) 24(5):879–80. 10.1016/j.ajt.2024.01.021 38266710

[21] Hope MD Raptis CA Shah A Hammer MM Henry TS. A Role for CT in COVID-19? What Data Really Tell Us So Far. Lancet (2020) 395(10231):1189–90. 10.1016/S0140-6736(20)30728-5 PMC7195087 32224299

[22] Peghin M Graziano E Grossi PA. SARS-CoV-2 Vaccination in Solid-Organ Transplant Recipients. Vaccines (Basel) (2022) 10(9):1430. 10.3390/vaccines10091430 36146506 PMC9503203

[23] Zavala S DeLaurentis C Aaron JG Miko BA Fox AN Bergelson M When You Need to Dive in the Deep End-Transplanting SARS-CoV-2 PCR+ Recipients. Transpl Infect Dis (2023) 25:e14110. 10.1111/tid.14110 37527176

[24] Nguyen LH Drew DA Graham MS Joshi AD Guo CG Ma W Risk of COVID-19 Among Front-Line Health-Care Workers and the General Community: A Prospective Cohort Study. Lancet Public Health (2020) 5(9):e475–e483. 10.1016/S2468-2667(20)30164-X 32745512 PMC7491202

