# Supplementary material for: Acceptance of Organs from Deceased Donors With Resolved or Active SARS-CoV-2 Infection: A Survey From the Council of Europe
Source: Transpl Int. 2024 Nov 21;37:13705. doi: 10.3389/ti.2024.13705 (PMC11617184; doi:10.3389/ti.2024.13705)
Supplement: Supplementary file 1 [file DataSheet1.docx]

**Supplementary Table 1. Organs considered for transplantation from donors with resolved and active SARS-CoV-2 infection**

|  | **Donors with resolved COVID-19**  n/N, % (n=28) | **Donors with active COVID-19**  n/N, % (n=20) |
| --- | --- | --- |
| Liver | 27 (96.4) | 19 (95) |
| Kidney | 28 (100) | 19 (95) |
| Heart | 25 (89.3) | 18 (90) |
| Lung | 18 (64.3) | 2 (10) |
| Bowel | 17 (60.7) | 6 (30) |
| Pancreas | 21 (75) | 15 (75) |

**Supplementary Table 2. Are recipients with active SARS‑CoV‑2 infection allowed to receive organs from donors with active SARS‑CoV‑2 infection?**

|  | n/N, % (n=21) |
| --- | --- |
| Yes, only if recipients are asymptomatic or with mild symptoms for heart and liver transplant | 1 (4.8) |
| Yes, only if recipients are asymptomatic or with mild symptoms for kidney, heart, and liver transplant | 1 (4.8) |
| No, they are excluded | 11 (52.4) |
| Case-by-case evaluation | 8 (38.1) |

**Supplementary Table 3. When can recipients with recent SARS-CoV-2 positivity be re-included on the transplant waiting list?**

|  | n/N, % (n=24) |
| --- | --- |
| After resolution of symptoms and documented virological cure with negative SARS-CoV-2 PCR | 19 (79.2) |
| After resolution of symptoms, with SARS-CoV-2 PCR documented virological cure not required | 3 (12.5) |
| After documented virological cure, with resolution of symptoms not required | 1 (4.2) |
| Case-by-case evaluation | 1 (4.2) |

**Supplementary Table 4. Vaccines protocols in waited listed patients**

| **Is SARS-CoV-2 vaccination mandatory for waited listed patients in your country?** | n/N, % (n=32) |
| --- | --- |
| Yes | 5 (15.6) |
| No | 27 (84.4) |
| **Is it planned to make it mandatory?** | n/N, % (n=27) |
| Yes | 0 (0) |
| No | 27 (100) |
| **What is the number of doses recommended in national protocols for waited list patients?** | n/N, % (n=26) |
| Minimum 1 dose | 0 (0) |
| Minimum 2 doses | 2 (7.7) |
| Minimum 3 doses | 17 (65.4) |
| Minimum 4 doses | 4 (15.4) |
| Minimum 5 doses | 3 (11.5) |

**Supplementary Table 5. Immunity screening in waited listed patients**

| **Is SARS-CoV-2 IgG measurement routinely performed in waitlisted patients?** | n/N, % (n=32) |
| --- | --- |
| Yes, at the registration on the waiting list | 1 (3.1) |
| Yes, at the registration on the waiting list and before transplantation | 6 (18.8) |
| Yes, at the registration on the waiting list, periodically while on the waiting list and before transplantation | 2 (6.2) |
| No | 21 (62.6) |
| Infectious disease specialist’s decision | 2 (6.2) |
| **Is SARS-CoV-2 virus-specific cell-mediated immunity routinely performed in waited list patients?** | n/N, % (n=30) |
| Yes, at the registration on the waiting list | 0 (0) |
| Yes, at the registration on the waiting list and before transplantation | 3 (10) |
| Yes, at the registration on the waiting list, periodically while on the waiting list and before transplantation | 0 (0) |
| No | 25 (83.3) |
| Infectious disease specialist’s decision | 2 (6.7) |

**Supplementary Table 6. Is a specific recipient informed consent required for patients receiving organs from a deceased donor with resolved or active SARS‑CoV‑2 infection?**

|  | n/N, % (n=25) |
| --- | --- |
| Yes, only from deceased donors with active SARS‑CoV‑2 infection | 11 (44) |
| Yes, only from deceased donors with resolved SARS-CoV-2 infection | 4 (16) |
| Yes, both from deceased donors with active and resolved SARS-CoV-2 infection | 2 (8) |
| No | 8 (32) |

**Supplementary Table 7. Tests and timing requested for screening of donors for SARS-CoV-2 infection**

| **Test required** | **Lung or bowel donation**  n/N, % (n=26) | **Non-lung non-bowel donation**  n/N, % (n=29) |
| --- | --- | --- |
| SARS-CoV-2 PCR in nasopharyngeal swab or lower respiratory tract sample | 3 (11.5) | 13 (44.8) |
| SARS-CoV-2 PCR in nasopharyngeal swab and lower respiratory tract sample | 18 (69.2) | 10 (34.5) |
| SARS-CoV-2 PCR in nasopharyngeal swab | 1 (3.9) | 5 (17.2) |
| SARS-CoV-2 PCR in lower respiratory tract sample | 4 (15.4) | 1 (3.5) |
| **Timing** | **Lung or bowel donation**  n/N, % (n=28) | **Non-lung non-bowel donation**  n/N, % (n=29) |
| 24 hours prior to organ recovery | 17 (60.7) | 18 (62.1) |
| 48 hours prior to organ recovery | 7 (25) | 8 (27.6) |
| 72 hours prior to organ recovery | 4 (14.3) | 2 (6.9) |
| > 72 hours prior to organ recovery | 0 (0) | 1 (3.4) |

**Supplementary Table 8. Is donor SARS-CoV-2 PCR Ct value required and considered in the decision-making process?**

|  | n/N, % (n=28) |
| --- | --- |
| Yes | 11 (39.3) |
| No | 14 (50) |
| Case-by-case evaluation | 3 (10.7) |

**Supplementary Table 9. Is SARS-CoV-2 IgG measurement and/or SARS-CoV-2 virus-specific cell-mediated immunity routinely performed in donors?**

|  | n/N, % (n=30) |
| --- | --- |
| Yes, both IgG measurement and SARS-CoV-2 virus-specific cell-mediated immunity | 1 (3.3) |
| Yes, SARS-CoV-2 IgG measurement | 2 (6.7) |
| Yes, SARS-CoV-2 virus-specific cell-mediated immunity | 0 (0) |
| Yes, IgG and SARS‐CoV-2 neutralizing antibodies | 0 (0) |
| No | 27 (90) |

**Supplementary Table 10. Analysis on donor samples**

|  | **In both donors with resolved and active COVID-19** | **Only in donors with active COVID-19** | **Only in donors with resolved COVID-19** | **No, only in specific setting** |
| --- | --- | --- | --- | --- |
| Is a SARS‑CoV‑2 result on biopsies or other donor samples required at the time of donation? n/N, % (n=26) | 1 (3.8) | 2 (7.7) | 0 (0) | 23 (88.5) |
| Is an analysis of donor organ quality by biopsy required for donation? n/N, % (n=27) | 0 (0) | 1 (3.7) | 0 (0) | 26 (96.3) |
| Is SARS‑CoV‑2 result on preservation fluid required at the time of donation? n/N, % (n=26) | 0 (0) | 0 (0) | 1 (3.8) | 25 (96.2) |

**Supplementary Table 11. Is chest imaging by CT scan routinely performed for donors with resolved and active SARS‑CoV‑2 infection?**

|  | n/N, % (n=28) |
| --- | --- |
| Yes, both for donors with resolved and active SARS‑CoV‑2 infection | 12 (42.9) |
| Yes, only for donors with active SARS‑CoV‑2 infection | 2 (7.1) |
| Yes, only for donors with resolved SARS‑CoV‑2 infection for lung donation | 4 (14.3) |
| No, only in specific setting | 10 (35.7) |

**Supplementary Table 12. During organ procurement and transplant, which infection control measures are recommended?**

|  | n/N, % (n=28) |
| --- | --- |
| Filtering facemasks (N95, FFP2 and FFP3) | 23 (82.1) |
| Eye protection | 19 (67.6) |
| Standard gloves and gowns | 28 (100) |
| Dedicated operating theatre | 7 (25) |
| Other | 0 (0) |

**Supplementary Table 13. In-hospital management of recipients of organs from donors with resolved and active SARS‑CoV‑2 infection**

|  | **Donors with resolved COVID-19**  n/N, % (n=26) | **Donors with active COVID-19**  n/N, % (n=17) |
| --- | --- | --- |
| Recipients are placed in isolation in an individual room in a non-COVID-19 area but with isolation procedures | 5 (19.2) | 5 (29.4) |
| Recipients are placed in isolation in a COVID-19 area | 0 (0) | 1 (5.9) |
| Recipients are not placed in isolation and are managed as routinely | 20 (76.9) | 10 (58.8) |
| Other | 1 (3.9) | 1 (5.9) |

**Supplementary Table 14. Vaccines protocols for health care workers and family members**

| **Is SARS-CoV-2 vaccination mandatory for health-care workers in your country? DOMANDA 36** | n/N, % (n=32) |
| --- | --- |
| No | 20 (62.5) |
| Yes | 12 (37.5) |
| **Is it planned to make it mandatory?** | n/N, % (n=20) |
| Yes | 0 (0) |
| No | 20 (100) |
| **Is SARS-CoV-2 vaccination mandatory for family members visiting hospitals in your country?** | n/N, % (n=32) |
| Yes | 0 (0) |
| No | 32 (100) |
| **Is it planned to make it mandatory?** | n/N, % (n=30) |
| Yes | 0 (0) |
| No | 30 (100) |

**Supplementary Table 15. Have you observed SARS-COV-2 infections among healthcare workers in your country related to the organ procurement or transplantation?**

|  | n/N, % (n=26) |
| --- | --- |
| Yes, in recipients of organs with resolved SARS‑CoV‑2 infection | 0 (0) |
| Yes, in recipients of organs with active SARS‑CoV‑2 infection | 0 (0) |
| No | 21 (80.8) |
| No available data | 5 (19.2) |

**Supplementary Table 16. Use of pre- and post-exposure prophylaxis when organs from donors with resolved or active SARS-CoV-12 infection are used**

|  | **In both recipients of organs from donors with resolved or active COVID-19** | **Only for recipients of organs from donors with resolved COVID-19** | **Only for recipients of organs from donors with active COVID-19** | **Only for recipients with active COVID-19** | **No** |
| --- | --- | --- | --- | --- | --- |
| Pre-exposure prophylaxis, n/N, % (n=27) | 0 (0) | 0 (0) | 0 (0) | 0 (0) | 27 (100) |
| Post-exposure prophylaxis, n/N, % (n=26) | 0 (0) | 2 (7.7) | 1 (3.8) | 1 (3.8) | 22 (84.6) |

**Supplementary Table 17.** **Is immunosuppression change suggested after transplant of organs from donors with resolved or active SARS-CoV-2 infection?**

|  | n/N, % (n=25) |
| --- | --- |
| Yes, both for recipients of organs from donors with resolved or active SARS‑CoV‑2 infection | 1 (4) |
| Yes, only for recipients of organs from donors with resolved SARS‑CoV‑2 infection | 0 (0) |
| Yes, only for recipients of organs from donors with active SARS‑CoV‑2 infection | 2 (8) |
| Yes, only for recipients with active SARS‑CoV‑2 infection | 3 (12) |
| No | 19 (76) |

**Supplementary Table 18.** **Have you observed SARS-COV-2 donor derived infections in your country/state?**

|  | n/N, % (n=25) |
| --- | --- |
| Yes, in recipients of organs from donors with resolved SARS‑CoV‑2 infection | 0 (0) |
| Yes, in recipients of organs from donors with active SARS‑CoV‑2 infection | 0 (0) |
| No | 25 (100) |
